# Health Tract, No. 129

**Published:** 1865

**Authors:** 


HEALTH TRACT, No. 129.

### BURNING TO DEATH.

THIS is a terrible calamity, yet it is a daily occurrence in any large city, and is almost always the result of gross carelessness, recklessness, or ignorance. Loss of life from the clothing taking fire may occur any hour in any family. The prevention and the remedy are matters of personal interest, at least to all parents; and certainly every school-teacher in the land should know how to act in the premises. Dresses can be made so that they will not readily take fire. The most available plan, the most economical and most accessible is to soak the clothing in strong salt-water just before wringing it out. There are other preparations used, such as a solution of sulphate of ammonia, tungstate of soda, etc., but the advantage of common salt is, that while it is as efficacious as others, it is not so liable to injure the colors of the dress. But it is not the wisdom of the times to prevent calamities. The next best thing is to know how to act in case of the dress taking fire. The beautiful and accomplished wife of a great name lately died, within the hour, by her dress having taken fire from a bit of blazing sealing-wax falling on it, while she was affectionately amusing her sweet little children at the sewing-table. Her husband was in an adjoining room and was instantly at her side, but either had not the knowledge or the presence of mind to arrest the progress of the flames. Perhaps three persons out of four would rush right up to the burning individual and begin to paw with their hands, without any definite aim. It is useless to tell the victim to do this or that, or call for water. In fact, it is generally best to say not a word, but tear up the carpet, or seize a blanket from the bed, or a cloak, or any woolen fabric—if none is at hand, take any woven material—hold the corners as far apart as you can, stretch them out higher than your head, and running boldly to the person, make the motion of clasping in the arms, most about the shoulders, this instantly smothers the fire and saves the face; the next instant throw the unfortunate on the floor; this is an additional safety to the face and breath, and any remnant of flame can be put out more leisurely. The next instant immerse the burned part in cold water, and all pain will cease with the rapidity of lightning. Next get some common flour, remove from the water and cover the burned parts with an inch thickness of the flour if possible. Put the patient to bed and do all that is possible to soothe, until the physician arrives. Let the flour remain until it falls off of itself, when a beautiful new skin will be found. Unless the burns are deep, no other application is needed. The dry flour for burns is the most admirable remedy ever proposed, and the information ought to be imparted to all; the principle of its action is that like the water, it causes instant and perfect relief from pain by totally excluding the air from the injured parts. Spanish Whiting and cold water of a mushy consistence is preferred by some Dredge on the flour until no more will stick, and cover with cotton batting. In washing clothes, use one part of sulphate of Ammonia with nine of water; one pound of tungstate of soda to a gallon of water. Dresses to be starched should have one third of tungstate and two thirds of starch.

